# The Impact of Differences in Surveillance Definitions of Hospital Acquired Urinary Tract Infections (HAUTI)

**DOI:** 10.3390/antibiotics10101262

**Published:** 2021-10-18

**Authors:** Yossef Levi, Debby Ben-David, Inna Estrin, Hodaya Saadon, Maya Krocker, Lili Goldstein, Dan Klafter, Shani Zilberman-Itskovich, Dror Marchaim

**Affiliations:** 1 Sackler School of Medicine, Tel-Aviv University, Tel-Aviv 6997801, Israel; yossi.levi15@gmail.com (Y.L.); debbybd@tlvmc.gov.il (D.B.-D.); shani.zilberman@mail.huji.ac.il (S.Z.-I.); 2 Unit of Infection Control, Wolfson Medical Center, Holon 5822012, Israel; 3 Unit of Infection Control, Shamir (Assaf Harofeh) Medical Center, Zerifin 7030000, Israel; innae@shamir.gov.il (I.E.); hodayas@shamir.gov.il (H.S.); mayakr@shamir.gov.il (M.K.); LiliG@shamir.gov.il (L.G.); dankl@shamir.gov.il (D.K.)

**Keywords:** surveillance, infection control, HAUTI, HAI, nosocomial infections

## Abstract

Hospital-acquired urinary tract infections (HAUTI) are common and most cases are related to catheters (CAUTI). HAUTI and CAUTI surveillance is mandatory in many countries as a measure to reduce the incidence of infections and appropriately direct the allocation of preventable resources. The surveillance criteria issued by the Israeli Ministry of Health (IMOH), differ somewhat from that of the U.S. Centers for Disease Control and Prevention (CDC). Our study aims were to query and quantify the impact of these differences. In a retrospective cohort study conducted at Shamir Medical Center, for calendar year 2017, the surveillance criteria of both IMOH and CDC were applied on 644 patient-unique adults with “positive” urine cultures (per similar definitions). The incidence of HAUTI per IMOH was significantly higher compared to CDC (1.24/1000 vs. 1.02/1000 patient-days, *p* = 0.02), with no impact on hospitalization’s outcomes. The agreement rate between methods was high for CAUTI (92%), but much lower for all HAUTI (83%). The major error rate, i.e., patients diagnosed with HAUTI per IMOH but had no UTI per CDC, was 31%. To conclude, in order for surveillance to reflect the relative situation and direct allocation of preventable resources based on scientific literature, the process should be uniform worldwide.

## 1. Introduction

Healthcare-associated infections (HAIs) are the leading cause of in-hospital death, resulting from a nosocomial acquired condition [1]. The Centers for Disease Control and Prevention (CDC) estimated that 1.7 million people per year in the U.S. develop HAI, and as many as 100,000 people die of HAI or due to its consequences [2]. According to the European Centers for Disease Prevention and Control (ECDC), each day, as many as 80,000 hospitalized European patients (i.e., one out of 18 patients) develops HAI [3]. In developing countries, HAI rates are even much higher [4]. In addition, HAIs are frequently reported to regulatory authorities and the incidence reported, serves as ‘pay per performance’ measure [5,6]. Therefore, reducing the incidence of HAI is important to facilities from several aspects: patients’ safety, quality of care, fiscally, and in terms of its reputation. Studies had demonstrated that by simply establishing and implementing a comprehensive process for HAI surveillance, it leads to a significant and sustained reduction in HAI rates, due to the Hawthorne effect [3]. HAI surveillance could also direct the expenditure and allocation of prevention resources, which are substantial to facilities and continuously grows (particularly since the COVID-19 pandemic [7]). HAI surveillance has now became a standard practice in many facilities worldwide [8]. 

Hospital acquired urinary tract infection (HAUTI) is a common HAI, and 75–95% of HAUTI are associated with urinary catheters (CAUTI) [9]. HAUTI (both CAUTI and non-CAUTI HAUTI) incidence is routinely monitored and reported to regulatory authorities and to the general public, and serves as a ‘pay per performance’ outcome measure in many countries (i.e., a payment model that offers financial incentives to healthcare providers for meeting certain performance measures) [10]. Since the surveillance definitions for HAUTI and CAUTI are of such paramount importance to hospitals, they should reflect as close as possible the “true” risk and burden to patients and to facilities. Regional, national, and international surveillance programs also create thresholds, and enables facilities to measure themselves in comparison to other facilities, as long as surveillance definitions are equal and uniform.

There are some methodological debates pertaining to HAUTI surveillance definitions and processes, and the extent to which it reflects the true burden to patients and facilities [11]. The most accepted platform is that of the CDC [12], which is revised periodically, but some argue the criteria are not broad enough [13] and different criteria is implemented in various countries [6]. In Israel, mandatory HAUTI surveillance is executed since 2014, in accordance to criteria issued by the Israeli Ministry of Health (IMOH) [14]. There are no specified or unique features in Israel, which necessitates different surveillance scheme, apart from the simple fact that the criteria were issued by different people who choose not to embrace the CDC criteria as is, but instead, to issue different criteria. In 2017, when this study was launched, there were a number of differences between the Israeli IMOH (IMOH) and the CDC surveillance definitions, as summarized in Table 1.

As depicted in the Table, there were several differences in 2017 surveillance criteria that might contribute to incremental rates if reported according to IMOH criteria, vs. different criteria that might contribute to incremental rates if reported according to CDC. Our study aims were therefore to compare the two sets of surveillance definitions and determine whether the rates reported from Israel are an overestimation or an underestimation compared to the more widely applied CDC criteria, and try to determine which criteria better reflect the “true” burden of HAUTI on both patients and facilities. 

## 2. Results

### Descriptive Analyses of the Study Population

During the study period, there were 651 patients with positive urine cultures as per CDC and/or IMOH identical microbiological defining criteria. After excluding seven patients with duplicate cultures, we applied both CDC and IMOH HAUTI surveillance criteria on 644 patients. There were overall 259 HAUTIs as per CDC (113 Symptomatic UTI [SUTI] and 146 CAUTI, 1.02 per 1000 patient days), and 314 HAUTIs as per IMOH (176 CAUTI and 138 non-CAUTI-HAUTI, 1.24 per 1000 patient days, risk difference compared to CDC of 0.2, CI-95% 0.03–0.4, *p* = 0.02). Table 2 depicts the characteristics of the patients who had HAUTI either according to CDC or according to IMOH criteria (n = 357).

The mean age of patients with HAUTI (either per CDC or per IMOH) was 68.4 ± 20 years, 72% were elderly, 52% were functionally dependent and 30% were cognitively impaired. The mean Charlson’s combined condition score was 3 ± 2.6, and 9% of the patients had chronic catheter prior to their index hospitalization. Over 44% of patients had acute kidney injury at the time of diagnosis. The most common pathogen was *Enterococcus faecalis*, followed by *Escherichia coli*, *Klebsiella pneumoniae*, and *Pseudomonas aeruginosa*. There were 18% patients with a multi-drug resistant organism (i.e., MDRO; most commonly extended-spectrum beta-lactamase producing Enterobacterales [ESBLs]) and the median time to initiation of appropriate antimicrobials for the entire population was 2 days (range 0–7 days). The outcomes of patients with HAUTI were poor, with over 35% mortality rate after 90 days, and functional deterioration among 50% of patients who survived the index hospitalization. However, none of the outcomes was statistically significant between patients with HAUTI per CDC vs. patients with HAUTI per IMOH per univariable analyses (bottom Table 1) and multivariable analyses (data not shown). Table 3 depicts the rates of HAUTI and CAUTI as per CDC and IMOH.

As depicted in Table 3, there were wide variations between the absolute numbers and rates of infections, as monitored according to the CDC vs. the IMOH criteria. There were 135 patients who had CAUTI both according to CDC and IMOH (92% of CDC cases vs. 77% of IMOH cases), and 81 patients who had a non-CAUTI HAUTI both according to CDC and IMOH (72% of CDC cases vs. 59% of IMOH cases). Out of 176 CAUTI cases per IMOH, 37 patients (21%) were diagnosed as not having UTI at all per CDC. Out of 146 CAUTI cases per CDC, only 11 patients (8%) were diagnosed as not having any UTI as per IMOH.

These variabilities were further reflected by looking at individual cases, i.e., not only the absolute numbers and rates as depicted in Table 3, but specifically analyzing the differences per each patient and how the patient was diagnosed as per the surveillance method (Table 4 and Table 5). In this case, 216 patients (83% of CDC cases and 69% of IMOH cases) had HAUTI both according to CDC and IMOH criteria. Table 4 list the rates of discrepancies and agreements analyses between the CDC vs. the IMOH surveillance process. For this analysis, the CDC was used as the ‘gold standard’ referral methodology. These rates of discrepancies and agreements are depicted for all HAUTI (Table 4), and only for the CAUTI cases (Table 5).

As depicted in Table 4 and Table 5, the very major error rate, i.e., the number of patients who had infection per CDC, but were not “captured” as having an infection per IMOH criteria, was relatively high for HAUTI (i.e., 17%, Table 4), but much lower for CAUTI (i.e., 8%, Table 5).

The reasons for discrepant diagnosis as per IMOH vs. CDC were then further analyzed. Among the 138 patients with non-CAUTI HAUTI as per IMOH, 81 patients did indeed had SUTI per CDC, but 57 patients did not had SUTI (only 59% essential agreement rate): i.e., 34 patients were diagnosed per IMOH but not per CDC because of the age over 65 years criterion (which was later removed from CDC criteria in 2021, see Table 1), 21 patients received “directed antimicrobial therapy” (a criterion present only in IMOH criteria, see Table 1) and two patients were classified as having a “present on admission” (POA) infection per CDC. Of the 113 patients with SUTI per CDC, 81 had non-CAUTI HAUTI per IMOH (72% essential agreement rate), and the reasons for discrepancy (32 patients) were due to infection on day 3 (30 patients) and due to the larger infection window as per CDC (2 patients).

When we analyzed the discrepant CAUTI events and agreement rates as per the two surveillance methodologies (Table 5), the differences between the IMOH and the CDC were narrower compared to the differences in rates of the non-CAUTI HAUTI/SUTI rates events (Table 4): i.e., 141 of 176 essential agreement rate per IMOH (80%), and 141 of 152 essential agreement rate per CDC (93%). The reasons patients were diagnosed with CAUTI per IMOH but not per CDC was due to the “direct therapy” additional criterion (20 patients. Agents are listed in Appendix A), dysuria captured as a criterion per IMOH among 10 patients who had catheters (not a CAUTI criterion per CDC, see Table 1)), and 4 patients who had a POA infection per CDC but did have CAUTI per IMOH. The reasons patients had CAUTI per CDC but not per IMOH was due to infection on day 3 (4 patients) and due to the prolonged infection window as per CDC criteria (4 patients).

## 3. Discussion

The process of HAUTI surveillance should lead to reduction of HAUTI incidence, rand help to guide the allocation of prevention efforts and resources [3]. Therefore, HAUTI surveillance is important and even mandatory nowadays in many countries and institutions. The most accepted platform for HAUTI surveillance is that of the CDC [12]. In Israel, the HAUTI surveillance criteria are issued by the IMOH [14], and are different on several aspects from that of the CDC (Table 1), but the different criteria are not due to any epidemiological or other unique factors which are different in Israel. The criteria are different because different people issued it, who chose not to embrace the CDC HAUTI criteria as is. However, the surveillance criteria of the IMOH for both bloodstream infections and surgical-site infections, are similar to CDC criteria [15,16], but for HAUTI it differ. For surveillance processes to be effective, it is important to compare the rates of infections, while measuring it in a uniform method. This could also control and direct appropriately the allocation of preventive resources, further contributing to HAUTI reduction. Our study goal was to analyze these differences in HAUTI surveillance criteria and quantify the possible impact that these differences might have on the overall reported rates, and on the “accuracy” in which it reflects patients’ outcomes. Of the criteria that are different between the CDC and the IMOH, we further analyzed the impact that each different parameter might have on the overall rates.

In a retrospective cohort study, conducted at a single center, 644 patient-unique adults with “positive” urine culture/s (i.e., the definition of a positive culture is identical per CDC and IMOH), were reviewed and surveillance definitions were applied in both methods on the same cohort of patients. As depicted in Table 2, the majority of symptoms’ defining diagnoses were fever and dysuria in both methods (72% and 31% per CDC, and 64% and 25% per IMOH, respectively). The cohort of patients with HAUTI per IMOH were older than patients with HAUTI per CDC (76 years per IMOH vs. 66 ± 22 years per CDC). This results from the exclusion of fever as a single defining symptom among patients over 65 years, per CDC criteria, in 2017. This criterion was later revised by the CDC in 2021 [15], which would lead to a substantial increment of the median age of patients with HAUTI per CDC. 

Overall, the incidence of HAUTI per IMOH (1.24/1000 patient days) was significantly higher compared to the incidence of HAUTI per CDC (1.02/1000 patient days), i.e., yielding 55 additional HAUTI cases [risk difference: 0.2, 95% CI: 0.03–0.4, *p* = 0.02]. As depicted in Table 3, there were 135 patients with CAUTI both per CDC and IMOH. This constitutes 92% of all CAUTI patients per CDC, but only 77% of all CAUTI patients per IMOH. This means that the vast majority of patients which are diagnosed with CAUTI as per CDC, are also captured as CAUTI patients if IMOH criteria are applied. However, there is a large portion of patients who will be diagnosed with CAUTI as per IMOH criteria but will not have CAUTI as per CDC. Moreover, many of these patients will not have any UTI at all per CDC (21%, Table 3). These same proportions were also evident among patients who had a non-CAUTI HAUTI as per IMOH: i.e., 21% would be diagnosed as not having any UTI at all if CDC criteria are applied (Table 3).

We further analyzed the disagreement rates per individual patients, i.e., not the overall prevalence and rates as depicted in Table 2, but the rates of infections after capturing the final diagnosis for each individual patient as per the CDC vs. the IMOH criteria (Table 4). Since our study aim was to analyze the situation from the “Israeli perspective”, i.e., to analyze the impact of the different local surveillance scheme in comparison to a method established elsewhere, the CDC was considered the “referral” surveillance method for this analysis. The essential agreement rate, i.e., the patients who were captured as having CAUTI per IMOH and also had CAUTI per CDC (the referral methodology), was again relatively high and satisfactory (92%). However, for all HAUTI cases per IMOH (includes both CAUTI and non-CAUTI HAUTI), the essential agreement rate with the CDC (includes CAUTI and SUTI [15]) was much lower (83%). This implies that CAUTI surveillance per IMOH do not confound considerably the rates that would be reported if CDC criteria were adhered to, but the non-CAUTI HAUTI surveillance per IMOH, results a statistically significant “over-diagnosis” rate in comparison to the CDC. When we looked at individual cases, this was mainly driven due to two factors: (1) the CDC criteria not allowing fever to be captured as a defining symptom among patients over 65 years of age. As previously mentioned, this criterion was later revised by the CDC [15]. (2) The additional IMOH surveillance criterion “directed antibiotic therapy” (agents are listed in Appendix A), which practically leads to the diagnosis of HAUTI among patients with a positive urine culture who were treated with certain antibiotics, despite not having any other defining sign or symptom of UTI. This criterion leads to the diagnosis of HAUTI among patients who will be diagnosed as having asymptomatic bacteriuria (ASB) per CDC. ASB is a common nosocomial complication, which leads to worse hospitalization’s outcomes including extended length of stay, *Clostridioides difficile* infections, emergence and spread of multi-drug resistant organisms, and in some analyses even increase in-hospital mortality [17]. Therefore, it is problematic for Israeli facilities to direct prevention efforts in order to reduce ASB rates, as it is not monitored nor measured, but captured many times as a HAUTI. As depicted in Table 4, the major error rate of HAUTI, i.e., patients who had HAUTI per IMOH but no UTI at all per CDC, was as high as 31%. The very major rate of HAUTI, which depicts the number of patients that were supposedly “missed” if they were screened according to the IMOH criteria instead of the CDC ‘referral criteria’, was 17% for HAUTI, and only 8% for CAUTI (Table 5).

When we queried the outcomes of patients diagnosed with HAUTI per IMOH (includes CAUTI and non-CAUTI HAUTI cases) vs. patients with HAUTI as per CDC (includes SUTI and CAUTI cases), the majority of outcomes were worse as per IMOH criteria, but the differences were statistically insignificant (Table 1). In multiple separate multivariable models, no outcome was independently associated with the surveillance scheme (data not shown). Therefore, despite higher rates of HAUTI and CAUTI as per IMOH, it was not reflected in worse patients’ outcomes. The fact that in 2021, the CDC revised their criteria [15], and now patients older than 65 years, who are febrile, can meet the CDC HAUTI definition, implies that the age span of HAUTI cases per CDC will increase substantially in future years, and the insignificant differences in outcomes that were captured in this study, will probably be abolished completely. If the IMOH surveillance scheme that resulted 55 additional cases of HAUTI was not correlated to any worse outcomes of patients, than the potential high error rates, resulting from the different surveillance processes, should be considered and discussed. First, the different surveillance scheme and the major variations in HAUTI rates, imply that Israeli facilities could not measure themselves in comparison to facilities from other countries. Second, Israeli facilities could not rely on the body of controlled evidence that is published in the scientific literature, which deals with HAUTI prevention, but are based on studies that used the CDC surveillance scheme. For example, as previously mentioned, the IMOH “directed antibiotic therapy” criterion (Appendix A) leads to the diagnosis of HAUTI among many patients with ASB per CDC and make it difficult for Israeli facilities to direct prevention efforts in order to reduce ASB rates, as it is not uniformly monitored. Adopting the CDC definitions may enable Israeli hospitals to compare rates with other countries, and the differentiation between ASB and HAUTI will assist in the implementation of appropriate prevention measures (e.g., focusing on antimicrobial stewardship versus improving aseptic techniques of insertion and/or management of urinary catheters). Another confounding IMOH criteria are the repeated infection timeframe (RIT) definition, which is defined as the entire hospitalization: e.g., a patient who developed HAUTI on the 4th calendar day, i.e., at the beginning of hospitalization, could not have another HAUTI even if events are completely separated (even by weeks). Moreover, the fact that dysuria can serve as a CAUTI defining IMOH criterion even among patients with catheters, is another confounding factor. 

Our study has several limitations, primarily associated with the retrospective chart review-based design. The study was also executed in a single Israeli center, which limit its generalizability to other facilities. Due to changes in CDC and IMOH criteria since 2017, the agreement analysis (Table 4 and Table 5) might not be updated but represent the differences according to the surveillance schemes relevant to 2017. To conclude, we think IMOH should revise the surveillance criteria to resemble exactly the CDC scheme, as being already executed in Israel for bloodstream infections [15] and for surgical-site infections [16]. The study did not aim to query which surveillance scheme is “better”, but it illuminates the potential consequences of implementing a different surveillance scheme, which is different and unique to what is implemented and practiced worldwide. 

## 4. Materials and Methods

We conducted a retrospective cohort study at the Shamir (Assaf Harofeh) Medical Center (SMC), an 877-bed university-affiliated institution in central Israel, for the calendar year 2017. The local institutional review board committee approved the study prior to its initiation. At SMC, the Infection Control Unit routinely performs HAUTI surveillance in all units, all year long, according to IMOH criteria. 

All adult (over 18 years) patient-unique positive urine culture samples that were obtained in 2017 on the 3rd calendar day and onward (to meet both CDC and IMOH criteria), were included. Of note, the defining microbiological criteria for a “positive urine culture” are identical as per CDC and IMOH [12,14]. HAUTI surveillance according to CDC [12] and IMOH [14] guidelines for 2017, were rigorously applied on this entire cohort of patients. The final study population consisted of patients who met either the CDC or the IMOH HAUTI definition. For each participant we captured demographics, background/chronic conditions, various exposures to healthcare, acute illness indices, microbiological data, therapeutic data, and various clinical, microbiological, and fiscal outcomes. The definition of MDRO included any of the following: methicillin-resistant *Staphylococcus aureus* (MRSA), vancomycin-resistant enterococci (VRE), *Pseudomonas aeruginosa* (non-susceptible to ≥3 classes of supposedly effective antibiotics), extended-spectrum β-lactamase producing Enterobacterales (ESBL), *Acinetobacter baumannii* (non-susceptible to ≥3 classes of supposedly effective antibiotics), and carbapenem-resistant or carbapenemase-producing Enterobacterales (CRE) [18].

Three major statistical analyses were performed with SPSS (IBM; v. 27) [19]: (1) Descriptive analysis to characterize each study population. (2) Univariable and multivariable (logistic regression) case-control analysis of predictors for HAUTI and CAUTI as per IMOH vs. CDC criteria. (3) Univariable and multivariable cohort analysis (logistic and Cox regressions), quantifying the impact of the surveillance method on clinical outcomes of individual patients: e.g., mortality parameters, length of hospital stay following the HAUTI among survivors of the index hospitalization, functional status deterioration, “acquisition” of MDROs, acute *Clostridioides difficile* infections (CDI) in the following three months, additional hospitalizations in the following three months, and discharge to a long term care facility (LTCF) after surviving the index hospitalization and being initially admitted from home.

## 5. Conclusions

In a large trial involving 644 patient-unique adults with a positive urine culture, significant variations in HAUTI diagnoses (and to a lesser extent CAUTI) were documented, after applying the IMOH surveillance criteria vs. the CDC criteria, but no variations were noted in patients’ outcomes. In order for a surveillance process to be effective, it is important to incorporate universal benchmarks and thresholds, and in addition, surveillance should direct the implementation of preventable measures that have established efficacy supported by the presence of controlled evidence. This could further contribute to HAUTI reduction and standardize the surveillance of HAUTI worldwide.

## Figures and Tables

**Table 1:** The differences between the IMOH and the CDC surveillance definitions.

Criterion	IMOH	CDC	Impact
HAI time definition	The urine culture should be obtained at the 4th calendar day since admission	The first sign or symptom starts at the 3rd calendar day since admission	HAUTI rates potentially increased according to CDC
Infection window	Positive blood culture with the same pathogen (as in the urine) could serve as a HAUTI defining criterion, if it was taken the day prior until the day that follows the urine culture date (i.e., an overall infection window of 3 days)	Positive blood culture with the same pathogen (as in the urine) could serve as a HAUTI defining criterion, if it was taken three days prior and up to three days following the urine culture (i.e., an overall infection window of 7 days)	HAUTI rates potentially increased according to CDC
Repeated infection timeframe (RIT)	The IMOH RIT criteria in 2017: the entire hospitalization, i.e., every growth of the same pathogen from the same hospitalization, will be attributed to the same HAUTI. If a different pathogen grows from a different urine culture, it could be defined as a different HAUTI if it meets the other criteria, regardless the RIT.	Any growth from urine within the CDC RIT of 14 days, either the same or a different pathogen, is attributed to the same HAUTI	HAUTI new cases rates might be increased according to CDC (very minor), but HAUTI re-infection rates are surely and profoundly increased according to IMOH. Of note, in 2019, following data extraction completion for this study, the IMOH had embraced the CDC RIT definition
Fever as a sole defining symptom among the elderly	Fever alone, at any age, could serve as a HAUTI defining criterion	Among patients older than 65 years, fever (above 38 °C) alone with no other symptom or sign suggestive of UTI, was not an eligible HAUTI defining criterion.	HAUTI rates potentially increased according to IMOH. Of note, this criterion was deleted later-on, in 2021, from the CDC criteria set, following study completion.
Dysuria	Dysuria could serve as a defining criterion even among patients with catheters	Dysuria could not serve as a defining criterion among patients with catheters	CAUTI rates specifically are potentially increased according to IMOH
Directed antibiotic therapy	Patients with a positive urine culture but with no other sign or symptom of UTI (i.e., ABUTI per CDC), who were treated with certain antibiotics (Appendix A) from the day prior to five days following the HAUTI defining culture date, and there was no other indication for the antibiotic therapy	Similar criterion does not exist	HAUTI rates are potentially increased according to IMOH
CAUTI definition	The catheter should be in place for at least two days from the culture date only (regardless the other defining signs or symptoms suggestive of HAUTI).	the catheter should be in place for at least two days on the date of event (i.e., the day that the symptoms had started or culture was withdrawn)	CAUTI rates specifically are potentially increased according to CDC
Defining symptoms under one year of age	Dysuria and Apathy will be considered as eligible defining symptoms, while the CDC does not consider these symptoms as suggestive of UTI.	Supra-pubic tenderness and Lethargy will be consider as eligible defining symptoms, while the IMOH does not consider these symptoms as suggestive of UTI.	Irrelevant to this paper as we enrolled only patients over 18 years of age.

Note: IMOH—Israeli Ministry of Health; CDC—Centers for Disease Control and Prevention; HAI—Healthcare associated infection; HAUTI—Hospital acquired urinary tract infection; RIT—Repeated infection timeframe; UTI—Urinary tract infection; CAUTI—Catheter associated urinary tract infection; ABUTI—Asymptomatic bacteremic urinary tract infection.

**Table 2:** Descriptive characteristics of study’s population (n = 357).

Parameter		CDC (n = 259)		IMOH (n = 314)	
		Frequency	Valid Percent ^1^	Frequency	Valid Percent ^1^
**Demographics**					
Age (years), mean ± SD or median (range)		66 ± 22		76 (19–97)	
Female gender		164	64%	192	62%
Elderly (≥65 years old)		160	66%	229	76%
Hospitalization division at culture date	Medicine	141	54%	197	63%
	Surgery	36	14%	44	14%
	Obstetrics & Gynecology	50	19%	36	11%
	Adult ICU of any type	32	12%	37	12%
Hospitalization division of infection acquisition	Medicine	74	36%	81	43%
	Surgery	31	15%	28	15%
	Obstetrics & Gynecology	48	23%	30	16%
	Adult ICU of any type	52	25%	51	27%
**Background medical status/conditions**					
Dependent functional status on admission		123	49%	163	53%
Reduced consciousness or cognitive impairment in background		69	27%	100	32%
Dialysis		7	3%	13	4%
Cahrlson’s scores	Weighted index comorbidity (mean ± SD)	3 ± 2.6		3 ± 2.5	
	Combined condition score (mean ± SD)	6 ± 3.6		6 ± 3.3	
	10 Year survival probability [percent, mean (range)]	0% (0–99%) IQR (0–77%)		0% (0–99%) IQR (0–29%)	
**Signs and symptoms suggestive of UTI**					
Fever (>38 °C)		184	72%	199	64%
Supra-pubic tenderness		9	4%	7	2%
Flank pain		5	3%	5	3%
Urgency		6	2%	5	2%
Frequency		13	5%	10	3%
Dysuria		81	31%	78	25%
**Urinary catheter parameters**					
Chronic catheter in background		26	11%	28	9%
Presence of a urinary catheter at the date of HAUTI event ^2^ or the day before		164	63%	215	68%
No. of catheterization days, median (range)		8 (0–269)		6 (0–269)	
**Acute illness indices**					
Reduced consciousness or cognitive impairment at acute illness		91	36%	126	41%
Acute kidney injury ^2^		102	41%	142	47%
**Antimicrobial therapy**					
Patient received on the date of event ^2^ and/or culture date, a “directed” antimicrobial therapy as per IMOH criteria and list of agents		134	55%	200	64%
Patient received on the date of event ^3^ and/or culture date, a “directed” antimicrobial therapy, because the physician aimed to target and cover the growth from urine in the prescribed antimicrobial regimen		76	50%	120	57%
Days to appropriate therapy (as per in-vitro susceptibilities), days, median (range)		2 (0–24)		1 (0–57)	
Received appropriate therapy (as per in-vitro susceptibilities) in less than 48 h		69	27%	98	35%
**Microbiology—Causative pathogens**					
Gram-positives	*Enterococcus* specie	96	37%	113	36%
	*Staphylococcus aureus*	4	2%	3	1%
Gram-negatives	*Escherichia coli*	89	34%	95	30%
	*Klebsiella pneumoniae*	34	13%	57	18%
	*Proteus mirabilis*	19	7%	10	3%
	*Enterobacter* specie	4	2%	5	2%
	*Serratia marcescens*	1	0.3%	4	1%
	*Providencia stuartii*	3	1%	3	1%
	*Citrobacter freundii*	1	0.3%	1	0.3%
	*Citrobacter koseri*	1	0.3%	1	0.3%
	*Morganella morganii*	0	0%	1	0.3%
	*Pseudomonas aeruginosa*	31	12%	38	12%
	*Acinetobacter* specie	3	1%	3	1%
	*Stenotrophomonas maltophilia*	1	0.3%	1	0.3%
Accompanied bacteremia		19	8%	24	8%
MDRO ^4^ overall		45	17%	63	20%
	VRE	0		0	
Type of MDRO	MRSA	1	0.4%	1	0.3%
	ESBL/AmpC producing Enterobacterales	34	13%	52	17%
	Carbapenem-resistant *Pseudomonas aeruginosa*	3	1%	3	1%
	*Acinetobacter baumannii*	7	3%	7	2%
**Outcomes**					
Total length of stay, days, median (range)		19 (2–279)		21 (3–279)	
Died during current hospitalization		53	20%	67	21%
Died in 14 days		63	24%	85	27%
Died in 90 days		85	33%	120	38%
Among survivors of the index hospitalization only	Length of stay after excluding dead	15 (2–140)		16 (3–140)	
	Functional status deterioration following the HAUTI	97	46%	144	52%
	Discharge to LTCF after being originally admitted from home	69	32%	99	36%
	Additional hospitalization in the following 3 months	83	39%	113	41%
	CDI post hospitalization (in 3 months)	8	4%	9	3%

Note: CDC—Centers for Disease Control and Prevention; IMOH—Israeli Ministry of Health; OR—Odds ration; UTI—Urinary tract infection; HAUTI—Healthcare associated urinary tract infection; SD—Standard deviation; ICU—Intensive care unit; MDRO—Multi drug resistant organism; VRE—Vancomycin resistant enterococci; MRSA—Methicillin resistant *staphylococcus aureus*; ESBL—Extended spectrum beta lactamase; LTCF—Long term care facility; CDI—*Clostridium difficile* infection. ^1^ Valid percent: Excluding missing cases from the denominator. ^2^ An acute rise in creatinine level (>1.7 mg/dL, or 1.5 times of baseline creatinine), or drop in estimated glomerular filtration rate (GFR) by >50%. ^3^ Date of event: the date that signifies the initiation of HAUTI: i.e., could be the date of the first symptom or sign that eventually led to the diagnosis of HAUTI, or the culture date if it preceded the first documentation of any sign or symptom of HAUTI. ^4^ MDRO refers to any of the following: (1) *Staphylococcus aureus* resistant to oxacillin (i.e., MRSA), (2) vancomycin-resistant enterococci (any enterococci, i.e., VRE), (3) any Enterobacterales which is resistant to any 3rd or 4th generation cephalosporin (e.g., ceftriaxone, ceftazidime, cefotaxime, cefepime), (4) any Enterobacterales with meropenem MIC > 1 (i.e., two or more), (5) *Acinetobacter baumannii*, (6) carbapenem-resistant *pseudomonas aeruginosa*, (7) *Stenotrophomonas maltophilia*.

**Table 3:** The number of patients with CAUTI and non-CAUTI HAUTI events as per IMOH vs. CDC surveillance criteria.

	CAUTI per CDC	SUTI per CDC	POA per CDC	Not UTI per CDC	Sum
**CAUTI per IMOH**	135	0	4	37	176
**Non-CAUTI HAUTI per IMOH**	0	81	2	55	138
**Not UTI per IMOH**	11	32	37	250	330
**Sum**	146	113	43	342	

Note: CAUTI—Catheter associated urinary tract infection; CDC—Centers for Disease Control and Prevention; SUTI—Symptomatic urinary tract infection POA—Present on admission; UTI—Urinary tract infection; IMOH—Israeli Ministry of Health.

**Table 4:** HAUTI events and agreement/disagreement rates as per the two types of surveillances.

	HAUTI per CDC (No.)	No HAUTI per CDC (No.)	Overall (No.)	Essential Agreement (%) ^a^	Error Rate (%) ^b^		
					Minor	Major	Very Major
**HAUTI per IMOH**	216	98	314	83%	24%	31%	17%
**No HAUTI per IMOH**	43	287	330	75%			
**Overall**	259	385					

Note. HAUTI—hospital acquired urinary tract infections; CDC—Centers for Disease Control and Prevention; IMOH—Israeli Ministry of Health; UTI—Urinary tract infection. ^a^ Essential agreement is the rate of patients with HAUTI per IMOH, out of all patients with HAUTI per CDC (i.e., the referral methodology). ^b^ Minor error is the rate of patients who did not had any UTI as per IMOH, but according to the referral surveillance method (CDC), did have either CAUTI, SUTI, or POA. Major error is the rate of patients who had HAUTI per IMOH but not per CDC; Very major error is the rate of patients who had HAUTI per CDC but not per IMOH, i.e., the HAUTI per the referral methodology (CDC) was falsely not captured by the IMOH surveillance method.

**Table 5:** CAUTI events and agreement/disagreement rates as per the two types of surveillances.

	CAUTI per CDC (No.)	No CAUTI per CDC (No.)	Overall (No.)	Essential Agreement (%) ^a^	Error Rate (%) ^b^		
					Minor	Major	Very Major
**CAUTI per IMOH**	135	41	176	92%	24%	23%	8%
**No CAUTI per IMOH**	11	457	468	92%			
**Overall**	146	498					

Note. CAUTI—Catheter associated urinary tract infections; CDC—Centers for Disease Control and Prevention; IMOH—Israeli Ministry of Health; UTI—Urinary tract infection. ^a^ Essential agreement is the rate of patients with CAUTI per IMOH, out of all patients with CAUTI per CDC (i.e., the referral methodology). ^b^ Minor error is the rate of patients who did not had any UTI as per IMOH, but according to the referral surveillance method (CDC), did have either CAUTI, SUTI, or POA. Major error is the rate of patients who had CAUTI per IMOH but not per CDC; Very major error is the rate of patients who had CAUTI per CDC but not per IMOH, i.e., the CAUTI per the referral methodology (CDC) was falsely not captured by the IMOH surveillance method.

## Data Availability

The data presented in this study are available on request from the corresponding author.

## Abbreviations

**HAUTI**	Healthcare-Associated Urinary Tract Infection
**CAUTI**	Catheter-Associated Urinary Tract Infection
**IMOH**	Israeli Ministry of Health
**CDC**	Centers for Diasease Control and Prevention
**UTI**	Urinary tract infection
**HAI**	Healthcare-Associated Infection
**ECDC**	European Centers for Disease Prevention and Control
**COVID-19**	Coronavirus disease 2019
**RIT**	Reapeat Infection Timeframe
**CI**	Confidence interval
**SD**	Standard Deviation
**ICU**	Intesive Care Unit
**MDRO**	Multi Drug Resistant Organisms
**MRSA**	Methicillin-Resistant *Staphylococcus Aureus*
**ESBL**	Extended-Spectrum Beta-Lactamase
**LTCF**	Long-Term Care Facility
**CDI**	Clostridium Difficile Infection
**GFR**	Glomerular Filtration Rate
**MIC**	Minimum Inhibitory Concentration
**SUTI**	Symptomatic Urinary Tract Infectin
**POA**	Present On Admission
**SMC**	Shamir Medical Center
**VRE**	Vancomycin-Resistant *Enterococcus*
**CRE**	Carbapenem-Resistant *Enterobacteriaceae*
**CPE**	Carbapenemase-Producing *Enterobacteriaceae*
**SPSS**	Statistical Package for the Social Sciences

## Appendix A

Directed antibiotic therapy as per the Israeli Ministry of Health criteria:

Cefepime

Ceftazidime

Ceftriaxone

Cefuroxime

Cephalexin

Ampicillin

Piperacillin

Piperacillin-Tazobactam

Ciprofloxacin

Levofloxacin

Ertapenem

Imipenem

Meropenem

Amikacin

Gentamicin

Nitrofurantoin

Trimethoprim-Sulfamethoxazole
